# Role of Endogenous and Exogenous Tocopherols in the Lipid Stability of Marine Oil Systems: A Review

**DOI:** 10.3390/ijms17121968

**Published:** 2016-11-24

**Authors:** Guadalupe Miroslava Suárez-Jiménez, Carmen María López-Saiz, Hugo Enrique Ramírez-Guerra, Josafat Marina Ezquerra-Brauer, Saul Ruiz-Cruz, Wilfrido Torres-Arreola

**Affiliations:** 1 Departamento de Biotecnología y Ciencias Alimentarias, Instituto Tecnológico de Sonora, 85000 Ciudad Obregón, Sonora, México; msuarez@guayacan.uson.mx (G.M.S.-J.); sruiz@itson.edu.mx (S.R.-C.); 2 Departamento de Ciencias Químico Biológicas, Universidad de Sonora, Blvd. Luis Encinas y Rosales s/n, 83000 Hermosillo, Sonora, México; 3 Ingeniería Ambiental, Universidad Estatal de Sonora, Unidad Académica Hermosillo, Ley Federal del Trabajo s/n, 83100 Hermosillo, Sonora, México; k_rmelita@hotmail.com; 4 Departamento de Investigación y Posgrado en Alimentos, Universidad de Sonora, Blvd. Luis Encinas y Rosales s/n, 83000 Hermosillo, Sonora, México; hguerra84@gmail.com (H.E.R.-G.); ezquerra@guayacan.uson.mx (J.M.E.-B.)

**Keywords:** tocopherols, antioxidant, fish oil, muscle, lipid oxidation

## Abstract

In marine organisms primarily intended for human consumption, the quality of the muscle and the extracted oils may be affected by lipid oxidation during storage, even at low temperatures. This has led to a search for alternatives to maintain quality. In this sense, antioxidant compounds have been used to prevent such lipid deterioration. Among the most used compounds are tocopherols, which, due to their natural origin, have become an excellent alternative to prevent or retard lipid oxidation and maintain the quality of marine products. Tocopherols as antioxidants have been studied both exogenously and endogenously. Exogenous tocopherols are often used by incorporating them into plastic packaging films or adding them directly to fish oil. It has been observed that exogenous tocopherols incorporated in low concentrations maintain the quality of both muscle and the extracted oils during food storage. However, it has been reported that tocopherols applied at higher concentrations act as a prooxidant molecule, probably because their reactions with singlet oxygen may generate free radicals and cause the oxidation of polyunsaturated fatty acids in fish oils. However, when tocopherols are included in a fish diet (endogenous tocopherols), the antioxidant effect on the muscle lipids is more effective due to their incorporation into the membrane lipids, which can help extend the shelf life of seafood by reducing the lipid deterioration that occurs due to antioxidant synergy with other phenolic compounds used supplements in fish muscle. This review focuses on the most important studies in this field and highlights the potential of using tocopherols as antioxidants in marine oils.

## 1. Introduction

Lipid oxidation (LO) is defined as the formation of free radicals and hydroperoxides from reactions between fatty acids and oxygen and is one of the main causes of seafood product quality deterioration, even at low temperatures. This reaction is responsible for odour and flavour development during oil or muscle storage [1], which can often influence final product acceptance or rejection. The fatty acids that are present in marine oil systems are highly susceptible to oxidation; therefore, efficient handling, storage and distribution of the product are required [2]. Refrigerating, icing and freezing are the main techniques used to prevent marine product deterioration; however, these techniques are often insufficient to maintain the integrity of these foods, especially on fat species [3]. Therefore, the addition of antioxidants has been employed as an alternative technology. These compounds (natural or synthetic) can help decrease oil and/or muscle deterioration in seafood products under different storage conditions [4]. Antioxidants are often directly applied in oils at very low concentrations, thereby avoiding changes in the sensory attributes of the food [5]. In muscle, however, the most commonly applied technique is to use packages that contain the antioxidant, which, under a controlled release, can migrate into the food during storage, thereby retarding lipid oxidation and protein denaturation [3]. Initially, the most common antioxidants used in the food industry were of synthetic origin, such as butylated hydroxytoluene (BHT), which efficiently retards the deterioration of the oil of marine systems during storage [3,6]; however, the direct addition of such products (synthetic antioxidants) to food is restricted in the food industry because of their harmful effects on consumers’ health [7]. In this sense, natural antioxidants have been used with favourable results. Tocopherols (α, β, δ and γ isomers), which are abundant and effective isomers of vitamin E, are oil soluble compounds whose main function is to maintain and protect biological membranes from peroxidation [8]. This natural antioxidant, which is used widely in the pharmaceutical, cosmetic and food industries, is found in plant oils, such as palm and sunflower; its action mechanism is to act as a hydrogen-donating antioxidant [9]. Tocopherol has been used in marine products in both endogenous and exogenous ways [10,11]. Its endogenous use refers to its use as a supplement in diet of cultured organisms, where its effect on product quality (mainly muscle) during post-harvest storage has been evaluated [11,12]. Furthermore, it has been demonstrated that tocopherol isoforms can synergize efficiently with other antioxidants, such as caffeic acid [13]. In contrast, tocopherols have been used both directly and applied in packing films in marine oils to decrease quality losses in postcapture storage, demonstrating that it is a potent antioxidant in the food industry [14,15]. Consequently, the aim of the present review is to collect the most relevant information about application of tocopherol via endogenous and exogenous mechanisms to efficiently prevent lipid oxidation and maintain the quality of different marine oil systems.

## 2. Lipid Oxidation in Marine Oil Systems

Marine oils are primarily composed of triglycerides, which contain a wide variety of fatty acids, classified as saturated, monounsaturated and polyunsaturated (PUFAs). There are two main families of PUFAs, depending on unsaturation degrees (ω-3 or *n*-3, and ω-6 or *n*-6). However, very small quantities of ω-6, mainly linoleic (C_18:2 *n*-6_) and arachidonic (C_20:4 *n*-6_) acids, are found in fish oils, while the ω-3 family, such as eicosapentaenoic (C_20:5 *n*-3_) and docosahexaenoic (C_22:6 *n*-3_), comprise the most abundant fatty acids in these organisms, receiving special attention because of the health benefits associated with their high levels of PUFAs (Table 1) [16]. The human consumption of these molecules is beneficial to human health because they have been associated with the reduction of cardiovascular disease risks, possess anti-inflammatory and autoimmune properties, and play an important role in brain development [17,18,19]. However, PUFAs are very susceptible to deterioration by oxidation during storage under different conditions, even at frozen temperatures, and their large degree of unsaturation and high affinity to oxygen generate volatile compounds such as alcohols, aldehydes and ketones, which cause unpleasant odours and flavours in the food [20].

### 2.1. Lipid Hydrolysis

**Figure 1:**
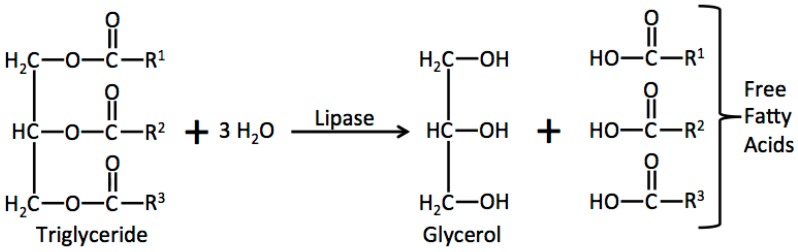
General scheme of enzymatic hydrolysis of triglycerides in marine oil systems.

Lipolysis occurs abundantly in marine oil systems (oil and muscle) during storage and is the main cause of quality deterioration. It is generally associated with the action of lipolytic enzymes, which is responsible for hydrolysing triglycerides, thus releasing fatty acids that undergo further oxidation producing oil rancidity (Figure 1) [26]. In fish muscle, it has been reported that lipid hydrolysis can continue even under refrigeration or frozen conditions due to the release of lipases from liposomes during the first storage stage [27,28]. Additionally, it has been reported that free fatty acids (FFA) correspond to less than 10% of the total fat content, which is increased significantly after freezing [2,29,30]. Although the formation of FFA is not considered a nutritional loss of the food, a prooxidant effect on lipids has been attributed to them; therefore, their release can be the start of rancidity development [31]. Additionally, FFA undergo a faster oxidation than do triglycerides due to their size differences [32].

### 2.2. PUFAs Oxidation

**Figure 2:**
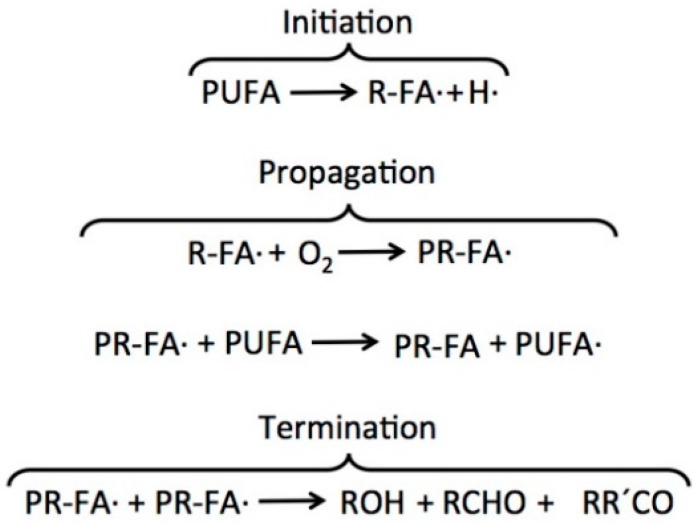
Main steps of PUFAs autoxidation in marine oil systems.

The formation of free radicals is the mechanism by which the oxidation of fatty acid polyunsaturated occurs [26]. A general scheme of PUFAs oxidation involves a process of initiation, propagation and termination (Figure 2). The initiation stage consists of a step in which a radical fatty acid (R-FA·) is produced by losing a hydrogen atom through an interaction with reactive oxygen species (oxygen ions, free radicals or peroxides, both inorganic and organic), and it can be accelerated by light or heat. In the second stage, propagation, the R-FA· (unstable molecule) rapidly reacts with molecular oxygen (O_2_) to create peroxyl radical fatty acid (PR-FA·), which can react with other PUFA, resulting in a different R-FA· (primary oxidation products) [33]. Finally, during termination, two radicals react and produce a non-radical species, such as alcohols, aldehydes and ketones; this occurs only when the R-FA· concentration is sufficiently high that the probability of a reaction occurring between two radicals increases. When a radical reacts with a fatty acid, it produces another radical via a chain reaction mechanism [34]. Lipid oxidation is a complex process, particularly due to the initiation, propagation and termination reactions, which can occur simultaneously [33].

### 2.3. Lipid Oxidation Consequences

Lipid oxidation in marine oil systems leads to a decrease in food quality. Although quality losses cannot be appreciated in the first stage of oxidation, during the next stages, they can be detected in flavour, colour, nutritional value and/or protein functionality [33]. The hydroperoxides produced during the propagation stage do not affect directly the flavour, odour or colour of food. However, the R-FA· decomposition into aldehydes and ketones causes changes in some characteristic sensorial attributes, which are commonly described as rancidity; this problem has a direct impact on consumer acceptance [33].

## 3. Role of Antioxidants

Antioxidants are generally classified as primary antioxidants, which react directly with free radicals and thereby inhibit the propagation step, or secondary antioxidants that inhibit the initiation and propagation reactions [33]. The main function of primary antioxidants is to donate hydrogens to the lipid free radical, which turns itself into a free radical. The antioxidant free radical then can react with other lipid peroxide radicals or other antioxidant free radicals to finish the reaction. Several primary antioxidants are endogenous in food systems, such as tocopherols, ascorbic acid, flavonoids, carnosine and glutation [20,33]. The effectiveness of primary antioxidants depends on their chemical structure, including their ability to donate electrons, and on their antioxidant radical stability [35].

Secondary antioxidants are oxygen liberators and chelators. Some examples of this type of compounds are superoxide dismutase, catalase, glutation peroxidase and carotenoids, which act by decreasing the active oxygen levels. Chelating agents include citrates, phosphates, ceruloplasmine and some free amino acids. It has been demonstrated that the concentrations of oxygen liberators and chelators decrease during icing storage [36]. Antioxidants are added directly to food; however, sanitary regulations restrict their direct use in some products, so there is special interest in incorporating them into the product packaging.

## 4. Effect of Antioxidants on Fish Muscle during Storage

Antioxidants (natural and synthetic) have been widely used to prevent lipid oxidation in fish muscle and extracted oils with different levels of effectiveness [3,5]. Their application, combined with frozen or chilling storage, can cause a significant increase in the product’s shelf life. However, the conditions in which the bioactive compounds are applied must be controlled: whereas very low concentrations may have no effect on the food, excessive concentrations may cause a pro-oxidant effect or undesirable changes in either the sensory and/or nutritional attributes of the stored product [14,20]. Initially, the use of antioxidants in seafood was evaluated on extracted oils following the direct application of low concentrations to the muscle, which showed an effective inhibition of lipid oxidation [4]; however, when applying these mechanisms, the antioxidant amount that migrates into the food during storage cannot be controlled. In this sense, the use of packaging is an alternative that provides a constant migration of the antioxidant to the food matrix [1,3,20]. The incorporation of the antioxidant into polyethylene films would be able to prevent lipid deterioration in marine oil systems [6]. Torres-Arreola et al. [3] tested the quality changes that appeared following the incorporation of BHT into a low-density polyethylene film used to cover sierra fish (*Scomberomorus sierra*) muscle and found that the use of this antioxidant can retard lipid oxidation and protein denaturation for 120 days at −20 °C, which indicated that the migration of the antioxidant towards the muscle can be carried out even during frozen storage. However, as noted, the use of synthetic antioxidants is restricted in some countries [20]. For this reason, in recent years, natural compounds have been applied to prevent lipid oxidation in marine oil systems [20].

## 5. Tocopherols as Antioxidants

As seen in previous studies, fish muscle is susceptible to different chemical reactions that lead to its deterioration, including oxidative reactions of both the lipids and proteins it contains [1,3,20]. Therefore, the use of compounds with antioxidant activity, such as tocopherols, in fish muscle is considered a viable option to delay these types of reactions.

### 5.1. Chemistry of Tocopherols

Tocopherols, also known as vitamin E due to their intervention in biological processes, are a group of well-known compounds that possess certain chemical characteristics that make them stand out as good antioxidants. These properties have been extensively reviewed; nevertheless, it is important to briefly describe them to better understand the effect that they can exert in a living organism such as fish.

Vitamin E was discovered in 1922 in a study on the influence of nutrition on rat reproduction [37]. During that research, the authors discovered that vitamin E was essential for reproduction; at that time, the vitamin was only known as α-tocopherol. The term Vitamin E later became a generic name given to all tocopherols (or tocols) and tocotrienol derivatives that are able to exhibit α-tocopherol biological activity [38].

**Figure 3:**
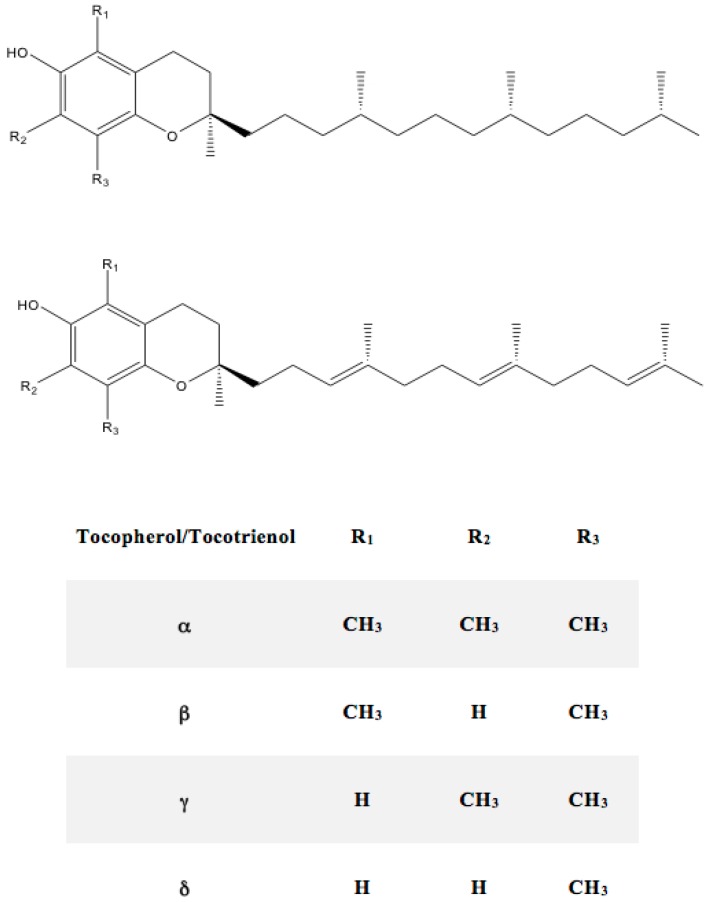
Chemical structures of the different types of vitamin E.

After its discovery, this group of compounds has been associated to different biological functions, such as membrane structure, prostaglandin synthesis, blood clotting, disease resistance and regulation of DNA synthesis [39]. The chemical structures of tocopherols and tocotrienols are represented in Figure 3. They are all composed of a 6-chromanol ring structure and a 16-carbon side chain [40]. This chain is saturated for tocopherols and unsaturated with three double bonds for tocotrienols at carbons 3, 7 and 11. The difference between the isomers tocopherols and tocotrienols lies in the position of the methyl substituents.

All Vitamin E components are fat-soluble; thus, it is considered a lipophilic vitamin. Although it is considered the main soluble lipid antioxidant in animals and its supplementation is recommended in human diets to prevent oxidative damage [41], it can be stored in animal tissues, so it does not have to be included in an everyday diet [42].

The biosynthesis of tocopherols can only be realized by photosynthetic organisms. The biosynthetic pathway was elucidated in the 1980s from the plastid isoprenoid, and this mechanism was reviewed by Raiola et al. [43]. The production of tocopherols inside a vegetable cell has been related to the plant’s response to oxidative stress [44].

### 5.2. Tocopherols’ Antioxidant Activity

The compounds known as Vitamin E, including both tocopherols and tocotrienols, are well recognized as antioxidants that exert their activity in foods and biological systems [38] and are incorporated into cellular membranes to inhibit lipid peroxidation [45].

The action mechanism of vitamin E consists of the donation of a phenolic hydrogen atom to a peroxyl radical, which converts it into a hydroperoxide. The tocopheroxyl radical produced is stable, and it cannot continue in the peroxidation cycle. Instead, it reacts with another peroxyl radical to form a non-radical product [46].

Vitamin E has been tested for its antioxidant activity in a variety of in vitro systems. An example is that of α-tocopherol, a compound known for its ability to scavenge free radicals in lipids [47] and can inhibit protein oxidation. It has been demonstrated that α-tocopherol can reduce the formation of α-aminoadipic and γ-glutamic semialdehyde from oxidized myofibrillar proteins [48].

Another example of a vitamin E antioxidant model is when the vitamin is incubated in vitro with platelets. In this case, lipid peroxide formation is reduced, and the activation of platelets is inhibited; thus, the vitamin can inhibit platelet aggregation [49].

### 5.3. Effects of Tocopherols in Living Organisms

In addition to the in vitro antioxidant activity, as is well known, vitamins are organic substances that are essential for different functions in living organisms and are required in small amounts.

An example of these effects was described by Al-Serwi and Ghomeim [50], who showed that vitamin E minimizes the toxicological effect of acrylamide in an in vivo rat study. The groups were treated with orally administered acrylamide and a dose of vitamin E, and the oxidative stress induced by acrylamide was reduced when the vitamin was administered.

The evidence of tocopherol’s antioxidant response in humans is still limited [51]; there are only a few studies related to the consumption of this vitamin with some beneficial health effects. Vitamin E has proven to reduce lipid peroxidation. In an in vivo study performed with 184 nonsmokers, to whom vitamins E and C were given as supplements to determine the effect of these vitamins on lipid peroxidation, the researchers found that both vitamins were able to reduce this phenomenon [52] due to their ability to donate either protons or electrons in an oxidation reaction.

Vitamin E, as reviewed by Colombo [42], was shown to inhibit cholesterol biosynthesis in animal cells through an enzyme suppression mechanism. In humans, when tocotrienols are administered as supplements along with lovastatin, which is a hypocholesterolemic agent, they are effective in reducing cholesterol, and the adverse effects of statins are avoided.

### 5.4. Vitamin E Content in Fish

Some fish species are not able to synthesize vitamins at all, and others can do so only in small quantities, which are not sufficient for the organism function. Therefore, they must be supplied in the fish diet. In this sense, marine mammals are highly dependent on adequate vitamin E consumption in their diet, which exerts some physiological functions, such as protecting their body tissues against oxidative stress [39]. As reviewed by Oliva-Teles [41], fish given food supplemented with vitamin E exhibited improvements in their immune response and disease resistance, whereas the vitamin administered with polyunsaturated fatty acids promoted a synergistic effect on that response.

Beneficial effects have been demonstrated in a variety of marine species; for example, when sea bass (*Dicentrarchus labrax*) is fed a vitamin E-supplemented diet, according to Obach A et al. [53], the fragility of their erythrocyte is diminished, as is the plasma lysozyme activity. When vitamin E was supplemented in a tilapia diet, it was proven to enhance reproductive performance; i.e., subjects were able to produce a large number of larvae in the individuals of this species [54]. In the case of beluga (*Huso huso* L.), researchers found that vitamin E supplementation helps marine organisms improve their weight gain and daily growth rate [55].

### 5.5. Tocopherols Bioavailability in Fish

The fraction of a consumed food substance that is available after ingestion is called bioavailability, and the one of vitamin E constituents depends on a number of different factors. The chemical compounds that constitute vitamin E are hydrophobic, therefore they require special transport mechanisms when present in aqueous environments such us body fluids, plasma and cells [56]. In humans it has been reported that vitamin E absorption is performed in the small intestinal lumen with the aid of biliary and pancreatic excretions where they are trapped in micelles and subsequently absorbed by intestinal epithelial cells by passive diffusion [57].

Tocopherols distribution among different tissues depends on its structure; in a research where both α- and γ-tocopherol were fed to Atlantic salmon (*Salmo salar*) [58], authors found that α-tocopherol was more deposited than γ-tocopherol on most tissues except in the perivisceral fat, and also they were stored on fatty tissues such as liver, serum, testes, kidney, brain, and gill. This could probably be due the presence of tocopherol transfer protein in the liver which is responsible for tocopherol binding and when the organism was subjected to tocopherol restriction, the main affected tissues were liver and muscle. Tocopherols that bind weakly to the tocopherol transfer protein are excreted in the bile [59].

### 5.6. The Role of Tocopherols in Cells

The function of the different constituents of vitamin E on fish cells includes the improvement of the immune system, decrease of lipid peroxides and reactive oxygen species production, up-regulate the activities of cytosolic phospholipase A2 as well as cyclooxygenase enzymes enhancing the release of prostacyclin, an inhibitor of platelet aggregation and a vasodilator [60]. Moreover, vitamin E provides a protection of highly unsaturated fatty acids in cells against oxidative degeneration, acting as a second line of defense peroxidative chain reactions scavenging fatty acyl peroxy radicals produced by this chain reaction [41]. Also, it has been demonstrated that α-tocopherol forms complexes with certain membrane components which have a tendency to destabilize the bilayer structure, countering their effects and making bilayer structures more stable [60].

### 5.7. Endogenous Applications of Tocopherols

In addition to the beneficial effect exerted by vitamin E on the biological and physiological functions on different fish species, from a food technology perspective, this vitamin helps improve the quality of muscle during storage. The antioxidant properties provided by their chemical structure are able to avoid some deterioration reactions.

One of the first reports of the enhanced antioxidative effect of tocopherols in fish supplemented with vitamin E is that of O’Keefe and Noble [11], who reported a reduction of oxidative reactions after frozen storage (−10 °C) on catfish (*Ictalurus punctatus*) supplemented with α-tocopherol; the longer the storage time is, the higher concentration the fish needs to be fed to ensure antioxidant activity. A few years later, this same organism was analysed; the author [10] discovered that the lipid oxidation products started to increase when tocopherols were degraded, which occurred approximately six months after frozen storage at −10 °C.

Another organism in which the antioxidant effect has been analysed is rainbow trout (*Oncorhynchus mykiss*). The levels of vitamin E reported in this species are in the range of 4.33–94.34 µg/100 g [61]. The fillets of trout supplemented with two different types of vitamin E were analysed when they were stored using both frozen and refrigerated conditions, and those with the higher vitamin E content had lower thiobarbituric acid-reactive substances [62].

In a different study involving three different marine species (*Scophthalmus maxiums* L., *Hippoglossus hippoglossus* L. and *Sparus aurata* L.), the authors found that when the fish were fed diets containing different amounts of vitamin E and a control group with no supplementation, the indicators of lipid peroxidation were highest in those fed the unsupplemented diet and in those fed the lowest vitamin E content. The authors thus concluded that a low vitamin E content in fish diets leads to higher levels of lipid peroxidation [63].

Red sea bream (*Pragrus major*) has also been studied, and an experiment was conducted on feeding oxidized fish oil to fish to determine the effects of different dietary vitamin C and E supplementation levels on fillet quality. The researchers found that vitamin C did not affect fillet quality but that the vitamin E concentrations reduced Thiobarbituric Acid Reactive Substances (TBARS) in the fillet. The authors suggested that this lipophilic vitamin could improve fillet oxidative stability in this species [64].

Wild Atlantic mackerel (*Scomber scombrus*) has also been studied. The stability of fatty acids and the amount of vitamin E on muscle during storage at −30 °C were measured, and the authors determined that neither the lipid content nor the fatty acid composition changed. Nevertheless, the quantity of vitamin E significantly decreased during the storage process, which indicated that vitamin E acted as an effective antioxidant [65].

By analysing these data, a few studies have suggested that monitoring tocopherol during storage might be a sensitive indicator of muscle stability prior to the formation of oxidation compounds [10].

Another aspect that must be considered when analysing the vitamin E concentration is that it varies among different types of muscle; the concentration of tocopherols in dark muscle has been found to be 4.4 times higher than that in light muscle [66]. Moreover, when different types of tocopherol are fed to marine species, the tocopherols deposit in different muscles and organs according to their chemical composition. For example, in a study on Atlantic salmon (*Salmo salar*), membranes with the highest content of phospholipids retained α-tocopherol better than γ-tocopherol, and these membranes are presumed to be the functional site for lipid antioxidants in vivo [58]. Therefore, this must be considered before choosing the appropriate tocopherol as a food supplement. In fact, according to Chan and Decker [67], the most efficient method to use γ-tocopherol as an antioxidant in skeletal muscle is through the diet. In this way, the compound is incorporated into the lipidic membrane, where the oxidation of skeletal muscle begins.

### 5.8. Exogenous Application of Tocopherols

Currently, the application of natural antioxidants such as tocopherols (which are classified as primary antioxidants) in fish oils and seafood-based products has increased in the food industry. Tocopherols are present in minor traces in bulk fish oils; however, trends toward the exogenous incorporation of these natural antioxidants have increased in the last several years. The beneficial properties of antioxidants have been described by some researchers. For example, unrefined mackerel oil treatments with α-tocopherol in concentrations of 50 and 100 parts per million (ppm) appear to be more effective in controlling oil oxidation than treatments with 250 and 500 ppm, with inhibition percentages of lipid oxidation around 75–80 and 55–60, respectively, after 66 days at −40 °C. This indicates that to obtain better antioxidant protection by α-tocopherol, lower concentrations are needed [14]. Moreover, the use of α-tocopherol in combination with low temperatures, i.e., refrigeration, contributes to the retardation of auto-oxidation kinetics in mackerel oil [14]. Similarly, Kul and Ackman [15] tested the antioxidant potential of α-tocopherol, applied at different concentrations (50–500 ppm), in unpurified menhaden oil and in a purified triacylglycerol (TAG) fraction; they observed a limited peroxidizing effect of α-tocopherol at the lower concentration used, whereas the initial formation rate of hydroperoxides was recorded at 100 ppm of α-tocopherol in both oils. Interestingly, the purified menhaden TAG was quickly oxidized in absence of α-tocopherol but was slightly more stable than the unpurified menhaden oil at the higher α-tocopherol concentrations [15].

As a consequence, a purified menhaden oil TAG was rapidly oxidized with no apparent induction period in an oxidation test conducted at 30 °C in darkness [15]. The ability of tocopherol isomers to retard the formation of hydroperoxides decreased in the following order: α > γ > δ at 100 ppm. However, an inverse antioxidant activity order of these molecules was observed at concentrations up to 1000 ppm [15].

Microencapsulation technology appears to be a suitable strategy to minimize auto-oxidation damage to marine oils [68]. The materials used in microencapsulation include vegetable and milk proteins, carbohydrates and biopolymers [69,70]; in several cases, these materials act as a barrier to atmospheric oxygen and free radicals, with two prooxidant factors in lipid systems. Even if no antioxidant is added prior to the microencapsulation of fish oils, positive effects on delaying oxidative damage can be gained by using this technology [68].

There are few reports regarding the use of tocopherols in microencapsulated fish oils as delivery systems. One study of this process involved fish oil, enriched milk and fish pate formulated with a microencapsulated fish/rapeseed oil mixture and showed good oxidative stability; these results were associated with the natural tocopherol content in rapeseed oil, which confers antioxidant protection to fish oil [68,71]. In contrast, Klinkesorn et al. [71] observed that tocopherol isomers incorporated at 500 ppm in tuna oil-in-water dried emulsions, which was stabilized with lecithin and chitosan via electrostatic layer-by-layer deposition technology, was effective in inhibiting TBARS on the order of 43%–45% at 37 °C for 13 days.

Spray-dried emulsions formulated with 37.5% *w*/*w* menhaden oil have also been studied. In this research, the application of α-tocopherol at 100 ppm was able to delay the onset oxidation period in refrigerated emulsion powders [72]. These emulsion powders had even lower peroxide values after three weeks of refrigeration storage than did those formulated with Trolox C when used at equal concentrations [68]. The lipophilic nature of α-tocopherol compared to that of Trolox C could be a key to understanding the difference in the antioxidant activity over the stability of menhaden emulsion powders. α-Tocopherol has a lipophilic side chain that facilitates its inclusion into oil-water emulsion interphases, but this characteristic is absent in Trolox C molecules, which have a hydrophilic chemical nature that is more associated with aqueous phases [72].

The use of tocopherols as natural antioxidants in post-harvest or post-catch handling in marine organisms has recently been explored. This is the case in shrimp, which is one of the most popular seafood items consumed around the world. The lipid amounts in shrimp muscle are lower than those of other commercial fish species such as mackerel, tuna, sardine, herring and anchovy [73]; however, some physicochemical and sensorial changes in shrimp quality could be related to either lipid deterioration or cross-linked reactions with other nutrients such as proteins and vitamins. In this sense, α-tocopherol’s antioxidant potential has been tested on frozen shrimps (*Litopenaeus stylirostris*) at −20 °C in two forms: as a glaze emulsion and as an antioxidant incorporated into bilayer polyamide-low density polyethylene films (PA-LDPE) [20]. In that study, long periods of both types of α-tocopherol application, glazed and antioxidant films, on frozen stored shrimps minimized lipid oxidation up to 90% over a period of 120 days [20].

### 5.9. Antioxidant Synergistic Effects of Tocopherols

Commonly, fish oils are consumed either as a whole or incorporated into a variety of processed foods such as pates, extruded snacks, energy bars, mayonnaise, yoghurt and salad dressings. These uses are due to the multiple healthy effects associated with their consumption [71,74].

Although fish oil incorporation into food products has recently been promoted, a variety of prooxidant factors may undergo in this matrix, thus compromising the oxidative stability of PUFAs and other nutrients. Because the prooxidant factors involved in lipid oxidation can greatly vary depending on the food matrix chemical composition, blends of antioxidants have been used to guarantee maximum protection on fish oil systems.

Some researchers tend to employ binary or ternary antioxidant blends food formulations with fish oils [75] because these blends can exert a synergic effect. Binary and ternary antioxidant systems have been evaluated in fish oils using α-, β-, δ- and γ-tocopherol isomer blends as well as combinations of α-tocopherol with ascorbyl palmitate and soy lecithins [15,75]. Such studies have investigated sardine skin lipids, where tocopherols were used in combination with lecithin and ascorbic acid. The results were compared to other antioxidants by measuring the oxidative stability [76]. α-Tocopherols only stopped the oxidation of sardine skin lipids for four days, which was less favourable than the results obtained for synthetic antioxidants (sodium erythorbate, BHA and TBHQ); however, a combination of α-tocopherols-lecithin-ascorbic acid was more efficient, extending the oxidation initiation stage to 14 days [76].

The antioxidant synergism of α-tocopherols with other chemical components was also studied by Yi et al. [77], who reported a cooperative interaction when tocopherol was used along with several compounds, including tocotrienols, carotenoids, ascorbyl palmitate and citric acid, thereby improving the lipid oxidative stability of fish/palm oleic oil mixtures.

The combination of a metal chelator such as ethylenediaminetetraacetate (EDTA) with tocopherols can also be effective in controlling the oxidative deterioration of tuna-oil emulsions by simultaneously controlling the prooxidant transition of metals and inactivating free radicals in emulsion droplets [25].

The oxidative stability of cod liver oil was tested along with γ-tocopherol, EDTA and ascorbyl palmitate, both individually and as a combination of all three antioxidants. When cod oil was incorporated into a salad dressing formulation at 10% *w*/*w* for nutritional purposes [71], the application of γ-tocopherol inhibited the PV values to 39% after six weeks compared to those observed without the antioxidant. Reports have shown that all three antioxidants exerted a significant impact on the oxidative stability of the enriched salad dressing, thus suggesting that more than one antioxidant mechanism was triggered, each associated with the characteristic chemical structure of a particular antioxidant [71]. Overall, a combination of γ-tocopherol/EDTA/ascorbyl (200/10/50 μg·g^−1^, respectively) completely inhibited the lipid oxidation of this salad dressing after six weeks of storage [71], suggesting a synergistic effect. However, specific oil systems have exclusive responses to specific antioxidant types and concentrations, occasionally including a prooxidant response. Therefore, in any given system, a number of stabilization schemes should be tested [78].

When marine oils are used in the food industry as either nutrients or emulsifiers in a variety of products, it is also crucial to consider the use of effective antioxidant schemes to guarantee the oxidative stability in each product. Moreover, tocopherol mixtures and their combination with other antioxidants such as metal chelators and vitamin C analogues are commonly used to ensure food product quality. However, the effectiveness of a specific antioxidant and/or a blend of antioxidants in a lipid system appear to be determined by multiple physicochemical factors that coexist in a complex food matrix. Regardless of the effectiveness demonstrated by certain antioxidant blends for the specific marine oil systems used in manufactured seafood based products, exceptions could occur. Therefore, the best way to select the appropriate antioxidant system is still by using experimental approaches.

## 6. Conclusions

This review highlights the importance of lipid oxidation in marine oil systems and the role played by the antioxidants that are used as a resource to prevent this phenomenon. Specifically, tocopherols have a proven ability to maintain food quality during storage under different conditions. Regardless of the way in which these natural antioxidants are applied (endogenous, exogenous, or in synergy with other antioxidants), tocopherols are highly effective in reducing lipid deterioration. However, their use in low concentrations and in synergy with other natural antioxidants has been demonstrated to exert greater effectiveness. In recent years, significant research contributions on the use of tocopherols to extend shelf life in marine origin organisms have been published, specifically methods designed to avoid those reactions related to lipid oxidation and protein denaturation. Nevertheless, more research efforts focused on the technological and nutritional aspects of marine origin foods are necessary due to the high-demonstrated potential of using tocopherols as antioxidants during the storage of marine oil systems. Moreover, further studies of the effect that tocopherols have on lipid-protein stabilization in stored fish muscle under different conditions are needed.

## Figures and Tables

**Table 1:** Polyunsaturated fatty acids (PUFAs) composition in different marine oils systems.

Marine Source	PUFAs Content (g/100 g Total Fatty Acids)	Reference
Atlantic Salmon (*Salmo salar*)	≈28	[21]
Atlantic menhaden (*Brevoortia tyrannus*)	≈26	[22]
Atlantic Cod (*Gadus morhua*)	≈22	[5]
Monterey sardine (*Sardinops sagax caerulea*)	≈25	[22]
Pacific sierra (*Scomberomorus sierra*)	≈28	[23]
Pacific corvina (*Cynoscion phoxocephalus*)	≈23	[23]
Pink Salmon (*Oncorhynchus gorbuscha)*	≈30	[24]
Tuna fish (*Thunnus*)	≈35	[25]
